# Bone Health in Metabolic Syndrome—Is It a Neglected Aspect of Dysmetabolic-Related Diseases?

**DOI:** 10.3390/jcm14165785

**Published:** 2025-08-15

**Authors:** Emilia Biamonte, Giulia Bendotti, Giulia Nigro, Beatrice Cavigiolo, Marco Gallo

**Affiliations:** 1 Endocrinology and Metabolic Diseases Unit, Azienda Ospedaliera Universitaria SS Antonio e Biagio e Cesare Arrigo, 15121 Alessandria, Italy; giulia.bendotti@ospedale.al.it (G.B.); marco.gallo@ospedale.al.it (M.G.); 2 Metabolic and Nutritional Disease Unit, ASST Bergamo EST, Ospedale Bolognini, 24068 Seriate, Italy; giulia.nigro@asst-bergamoest.it; 3 Endocrinology and Metabolic Diseases Unit, ASL Vercelli, 13100 Vercelli, Italy; beatrice.cavigiolo@aslvc.piemonte.it

**Keywords:** bone health, osteoporosis, fracture risk, mineral density, trabecular bone score, insulin resistance, metabolic syndrome, obesity, dyslipidemia, hypertension

## Abstract

Due to their widespread prevalence and the aging global population, metabolic syndrome (MetS) and osteoporosis represent significant public health challenges. Clinical interest in MetS is currently primarily focused on cardiovascular risks. However, emerging evidence indicates that metabolic conditions may also adversely affect bone health. Each component of MetS—especially glucose metabolism impairment, central obesity, and endocrine factors—impacts bones in distinct ways, creating a complex network of interactions that influences skeletal health. These metabolic disturbances can lead to changes in bone remodeling, potentially resulting in alterations to bone mineral density and microarchitectural structure and an increased risk of fractures. Regarding uncertain and controversial pieces of evidence about the effect of MetS on bone health, this narrative review discusses and summarizes the current research on the association of MetS and its components with bone metabolism, bone quantity (based on bone mineral density, or BMD), bone quality (based on trabecular bone score, or TBS), and fracture risk.

## 1. Introduction

Osteoporosis (OP) and metabolic syndrome (MetS) represent two major and growing global public health challenges that contribute substantially to morbidity, mortality, and healthcare costs [1,2].

OP is a chronic skeletal disorder characterized by reduced bone mass and deterioration of bone microstructure, leading to an increased risk of fragility fractures. Affecting hundreds of millions of people worldwide, it is responsible for millions of fractures every year, with one osteoporotic fracture occurring approximately every three seconds [1,3]. Concurrently, MetS is defined by a combination of metabolic abnormalities, including central obesity, hypertension, hyperglycemia, and dyslipidemia as shown in Table 1. This prevalent disorder affects more than 20% of the general population with a growing trend globally [2]. While the strong association between MetS and cardiovascular diseases and type 2 diabetes is well-established, its precise impact on bone health remains an area of ongoing investigation and considerable debate [2,4]. Some evidence suggests that MetS and abdominal obesity may provide osteoprotection due to mechanical loading, which is associated with higher bone mineral density (BMD) and, consequently, a lower fracture risk [5,6]. Conversely, other studies indicated that the pro-inflammatory environment inherent to MetS, along with altered hormonal and biochemical profiles, may be detrimental to the bone, resulting in lower bone density and quality as well as a higher incidence of osteoporotic fractures [7,8].

Considering the uncertain and controversial evidence on the effect of MetS on bone health [6,7,8], this review aims to synthesize the current research on the association of MetS and its components with bone metabolism, bone quantity (based on BMD), bone quality (based on trabecular bone score, or TBS), and fracture risk.

### Methods

For this narrative review we thoroughly searched PubMed, MEDLINE, and Embase databases using the following keywords that were variously combined: “metabolic syndrome”, “bone health”, “osteoporosis”, “fracture risk”, “mineral density”, “trabecular bone score”, “hypertension”, “dyslipidemia”, “obesity”, and “insulin-resistance”. The abstract of every retrieved publication was screened, and, if potentially eligible, its full text was assessed by two of the authors (EB and GB). If a consensus could not be reached, a third reviewer (MG) was consulted. Existing reviews and the reference list of the selected studies were also consulted to identify any undetected report.

## 2. Bone Health in Metabolic Syndrome

**Figure 1:**
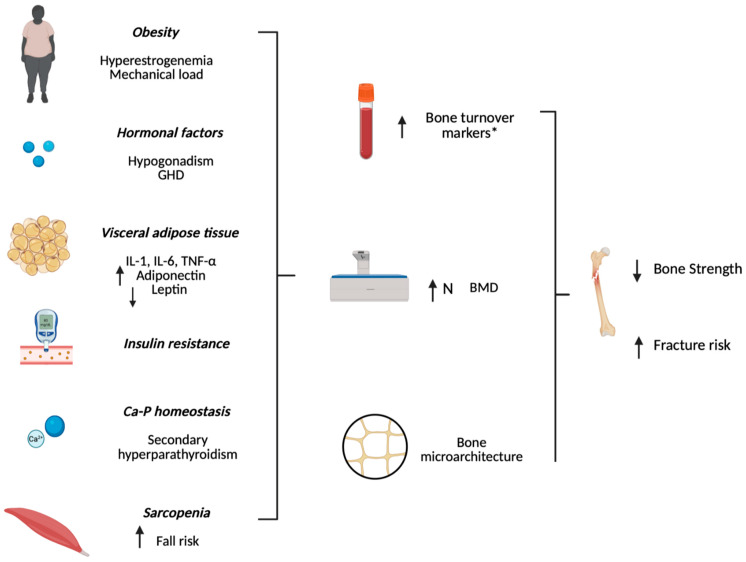
Created with BioRender.com. Pathogenesis of bone fragility and fracture in Metabolic Syndrome. Legend: GHD, growth hormone deficiency; IL-1, interleukin-1β; IL-6, interleukin-6; TNF-α, tumor necrosis factor-α; N, normal. * in insulin resistance; The ↑ symbol indicates an increase, and ↓ indicates a decrease.

**Figure 2:**
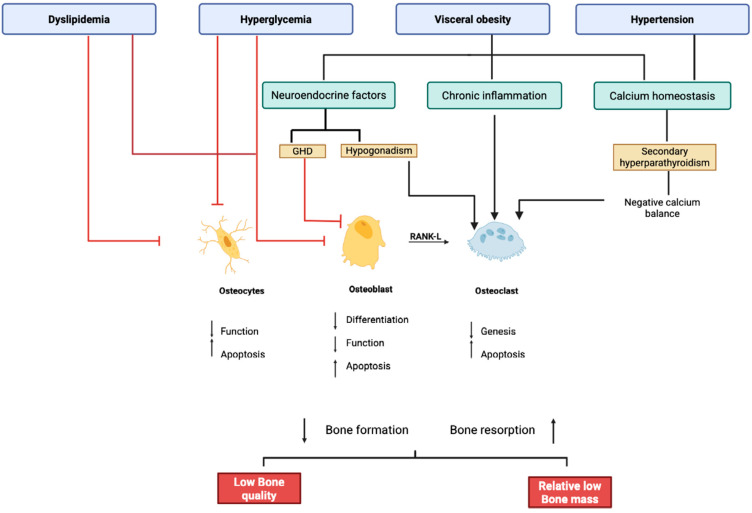
Created with BioRender.com. Impact of Metabolic Syndrom and its components on bone metabolism. Legenda: GHD, growth hormone deficiency; RANK-L, Receptor Activator of Nuclear Factor κB ligando.

The interplay between the components of MetS significantly influences bone health and fracture risk, particularly through the effects of central obesity, glucose metabolism impairment, and various endocrine factors. Key contributors to this interplay are illustrated in Figure 1, whereas the possible molecular mechanisms are illustrated in Figure 2.

Traditionally, obesity was considered protective against osteoporosis due to the positive correlation between body mass index (BMI) and bone mineral density (BMD) [9]. The higher BMD in obese individuals was often attributed to the mechanical loading from excess weight, which promotes skeletal adaptation through the reduction in sclerostin expression by osteocytes [10] and also hyperestrogenemia resulting from increased aromatase activity in adipose tissue [11]. However, the relationship between obesity and bone is quite intricate.

While mechanical loading and estrogen excess generally promote bone anabolism [10,11], it is now well acknowledged that the detrimental metabolic effects of obesity arise more from fat distribution than from the quantity of fat itself [12]. Visceral adipose tissue (VAT), in particular, is closely linked to insulin resistance (IR) and type 2 diabetes mellitus (T2DM) and produces biologically active substances that can adversely affect bone health. Indeed, VAT is inversely related to BMD, because it releases inflammatory cytokines such as interleukin-1β (IL-1β), interleukin-6 (IL-6), and tumor necrosis factor-α (TNF-α). These cytokines promote osteoclast differentiation and enhance bone resorption through activation of the RANKL/RANK/osteoprotegerin (OPG) pathway [13]. Adipokines, like leptin, typically increased in obesity, and adiponectin, tendentially reduced in obesity, are crucial players in this intricate interaction. Moreover, VAT further compromises bone integrity by influencing cortisol metabolism via 11β-hydroxysteroid dehydrogenase type 1 (11β-HSD1) [14].

Obesity is frequently accompanied by reduced muscle strength. This is due to the infiltration of muscle by adipose tissue, a condition known as myosteatosis, which adversely affects muscle structure and strength, leading to sarcopenic obesity [15]. The underlying mechanisms include adipose tissue pro-inflammatory actions, mitochondrial dysfunction, and oxidative stress [15,16]. Sarcopenic obesity significantly increases the risk of frailty, falls, and fractures, even counteracting any potential protective effect of obesity on the bone at certain sites [15,16].

Endocrine dysregulation is another critical factor. Obesity is associated with dysregulation of the GH/IGF-I and gonadal steroid axes, both crucial for bone homeostasis [17,18]. Visceral obesity can lead to a condition of relative GH deficiency (GHD) [17], which is associated with reduced bone mass and increased fracture risk, since GH stimulates osteoblast differentiation [17] and IGF-1 influences cortical bone structure, a major determinant of bone strength [19]. Moreover, GHD plays a significant role in modulating body composition, increasing abdominal adiposity, and decreasing muscle mass [19]. Additionally, obese males often exhibit reduced testosterone, a positive determinant of BMD and muscle mass, while estrogen production increases with body weight in both sexes [18].

The relationship between IR and bone is complex and not yet fully understood. While insulin generally has anabolic effects on bone, chronic hyperinsulinemia may paradoxically impair bone remodeling by reducing osteoblast proliferation and survival, while promoting osteoclast activity by upregulating RANK expression [20,21]. Moreover, the accumulation of advanced glycation end-products directly disrupts collagen properties and bone strength [22].

Less is known about the precise impact of hypertension and dyslipidemia, common MetS components, on the bone. However, observational studies suggest a link between hypertension and osteoporosis [23], potentially involving the renin–angiotensin system. Angiotensin II may induce RANKL expression in osteoblasts, promoting bone resorption [24]. Conversely, angiotensin receptor blockade has also been associated with a decreased incidence of bone fracture [24]. In dyslipidemia, particularly oxidized LDL may suppress osteoblast differentiation while promoting adipocyte formation in bone marrow, potentially leading to fatty marrow and reduced bone mass [25].

Importantly, current pharmacological interventions for MetS (Table 2) have shown potential effects on bone metabolism and/or fracture risk [26,27,28,29,30,31,32].

## 3. Bone Turnover

Measurement of biochemical bone turnover markers may be useful in the clinical management of skeletal fragility in patients with MetS. During bone formation, osteoblasts secrete the precursor of type 1 collagen, i.e., procollagen type 1 N-propeptide (P1NP), and osteocalcin, which are considered reliable markers of osteoblast function [33]. On the other hand, carboxyterminal cross-linking telopeptide of type 1 collagen (CTX-1) is released during bone resorption, designating it as reference marker of bone resorption in clinical practice [33]. Recent evidence highlights a complex and often contradictory relationship between MetS and bone turnover. In male rats subjected to a high-carbohydrate, high-fat diet, Wong et al. observed reduced osteoblast activity and elevated CTX-1 levels, indicating enhanced bone resorption despite no significant change in total BMD [34]. Similarly, human studies suggest that metabolic abnormalities of MetS may impair bone remodeling by promoting an imbalance between bone resorption and formation. Holloway-Kew et al. and Laurent et al. demonstrated that hyperglycemia is associated with suppressed bone turnover [35,36]. In the Geelong Osteoporosis Study, men and women with impaired fasting glucose (IFG) had significantly lower levels of CTX-1 and P1NP compared to those with normoglycemia, suggesting reduced bone resorption and formation [36]. This effect was particularly evident in younger participants, indicating early metabolic alterations in bone metabolism.

Also, obesity is negatively associated with bone turnover markers. Studies reported that obesity was associated with decreased levels of both formation and resorption markers, indicating an overall suppression of bone turnover [37,38]. These findings suggest that excess adiposity may impair skeletal remodeling, possibly through mechanisms involving IR and adipokine signaling. In contrast, hypertension alone was not significantly associated with changes in bone turnover markers [37]. Furthermore, Fodor et al. and Terzi et al. reported significantly reduced serum osteocalcin and CTX-1 in postmenopausal women with MetS compared to controls [39,40]. Despite higher BMD in MetS patients, the difference disappeared after adjusting for age and BMI. Both studies support a potential link between impaired glucose metabolism and reduced bone turnover, implying that MetS may affect bone quality despite preserved or increased density. While the predictive role of bone turnover markers in fracture risk remains uncertain, these findings indicate they may still be helpful in evaluating how metabolic syndrome affects bone metabolism.

## 4. Calcium–Phosphate Homeostasis

Calcium and phosphate disorders, along with vitamin D deficiencies, are increasingly recognized as crucial contributors to the complex interplay between MetS and bone health, influencing bone remodeling and fracture risk. Notably, IR, hypertension, and low-grade systemic inflammation, in particular, may affect the endocrine regulation of mineral metabolism [41,42].

Interestingly, some epidemiological studies have reported statistically lower serum calcium levels in MetS patients, albeit missing evaluation on dietary calcium intake [43]. Furthermore, individuals with various MetS components often exhibit lower circulating phosphate levels, likely mediated by elevated fibroblast growth factor 23 (FGF23). FGF23 plays a critical role by suppressing calcitriol (active vitamin D) synthesis through 1-alpha-hydroxylase inhibition, promoting phosphate excretion, and reducing its reabsorption. These actions collectively favor high bone turnover and can compromise bone microarchitecture [44].

Poor glycemic control, a hallmark of MetS, has also been observed to correlate with increased urinary calcium loss and subsequent secondary hyperparathyroidism [43]. This hyperglycemia-induced hypercalciuria could be mediated through glycosuria, with defective reabsorption of both glucose and calcium in the proximal tubule or collecting duct [45,46].

Similarly, hypertension can contribute to calcium–phosphate dysregulation through increased calcium excretion, often related to both high sodium intake and abnormalities in the renal tubular transport of sodium and calcium [44,45,47]. Moreover, antihypertensive drugs, such as loop diuretics, thiazide diuretics, and beta-blockers, may further contribute to this complex interacting network [43] as shown in Table 2.

Concurrently, the pro-inflammatory milieu associated with MetS, characterized by elevated TNF-α and IL-6, directly interferes with intestinal calcium absorption and suppresses renal 1-alpha-hydroxylase activity, thereby reducing calcitriol synthesis [41,48]. This leads to decreased intestinal absorption of both calcium and inorganic phosphate and contributes to relative hypocalcemia, which in turn stimulates PTH secretion and consequently enhances bone resorption.

Finally, hypovitaminosis D is a common condition in MetS, especially in obese individuals. Beyond its well-known role in calcium–phosphorus metabolism, vitamin D may regulate beta-cell pancreatic function and improve IR through direct and indirect mechanisms [49]. The vitamin D receptor (VDR) and the 1-alpha-hydroxylase-converting enzyme expressed in pancreatic beta cells suggests a direct impact on insulin secretion, where vitamin D insufficiency could interfere with normal insulin release by altering calcium flux. VDRs are also expressed in insulin-sensitive tissues like bone, muscle, and adipose tissue. Furthermore, vitamin D inhibits the activation of the nuclear factor NFKb and the expression of other cytokines that are harmful to beta cells [48,50]. Noteworthily, a recent meta-analysis demonstrated that calcium and vitamin D supplementation significantly improved MetS markers such as fasting glucose levels, insulin levels, and HOMA-IR [51]. Indeed, the disruption of mineral homeostasis in MetS creates a biochemical environment conducive to impaired bone remodeling and increased skeletal fragility. This underscores the need for early identification and targeted interventions in at-risk populations.

## 5. Bone Mass—Bone Mineral Density

BMD measured at the lumbar spine, total hip, and femoral neck by dual-energy X-ray absorptiometry (DXA) is the gold standard for the diagnosis of osteoporosis [52]. For postmenopausal women and men over 50 years old, a T-score below −2.5 indicates osteoporosis, while for premenopausal women and men under 50, a Z-score below −2.0 suggests below-average BMD for their age [52]. However, BMD alone is often insufficient to predict fragility fractures, particularly in secondary osteoporosis, where patients may have an elevated fracture risk despite normal BMD.

The relationship between MetS and BMD is complex and not fully understood, with numerous studies yielding inconsistent results. MetS includes conditions such as hyperglycemia, hypertension, dyslipidemia, and overweight/obesity, which may impair bone microarchitecture, even when BMD appears preserved.

Initially, several studies observed a positive association between MetS and BMD, showing higher T-scores at the femoral neck or lumbar spine in individuals with MetS compared to those without [53,54]. For instance, a large Korean study on post-menopausal women noted a slower rate of BMD loss over three years in MetS subjects [55]. Abdominal obesity emerged as the MetS component most strongly associated with osteoporosis. Indeed, the apparent positive relationship between bone mass and MetS is often attenuated or even reversed when BMD is adjusted for BMI or, more accurately, waist circumference (WC), a better body composition indicator [56]. Some research, adjusting for body weight and BMI, showed lower femoral neck BMD in men and postmenopausal women with MetS. Additionally, MetS seems to exert a more detrimental effect on men’s BMD than on women’s [56,57]. Studies that adjusted BMD based on WC revealed a higher prevalence of osteoporosis in individuals with larger waist sizes [58]. Concordantly, the Bushehr Elderly Health (BEH) program similarly identified an initial positive relationship between BMD and MetS [57]. However, this association lost significance after adjusting for individual components of MetS, such as abdominal obesity (measured by WC), high triglyceride levels, high blood pressure, and hyperglycemia. Despite these adjustments, BMD often remained higher in MetS subjects at certain skeletal sites, suggesting that, while MetS might be linked to a higher T-score, its components negatively impact bone quality.

The role of insulin and glucose metabolism on bone health is particularly complex. Many studies suggest that hyperinsulinemia and hyperglycemia may mediate an increase in BMD, independently of age, BMI, and waist–hip ratio. However, in stark contrast to these potential anabolic effects, IR has been linked to detrimental outcomes. For instance, in non-diabetic elderly individuals, even after accounting for higher BMD and BMI, higher IR has been associated with an increased risk of fracture [58].

This discrepancy between bone mass and fracture risk in MetS highlights an important point. Similar to other secondary osteoporosis, individuals with MetS may also have a relative deficiency in bone mass and abnormal bone microarchitecture. Although DXA is a primary diagnostic tool, it has limitations in this setting. Measurement of BMD in patients with obesity (BMI > 37 kg/m^2^) can be challenging, leading to inaccuracies due to increased fat mass and its distribution [59]. Furthermore, DXA does not differentiate between cortical and trabecular bone, and many metabolic diseases primarily affect trabecular bone microarchitecture, potentially resulting in a falsely normal BMD value. To obtain a more comprehensive understanding of a patient’s true fracture risk, it is recommended to evaluate the T-score in conjunction with the trabecular bone score (TBS) and a fracture risk assessment tool, such as the FRAX score.

Regarding hypertension and dyslipidemia, their precise relationship with osteoporosis is less clear. Mussolino et al.’s analysis of NHANES data found no significant association between BMD and hypertension in African American and white men and women [22]. Furthermore, a meta-analysis by Ye et al. showed a negative association between blood pressure and BMD and a positive association with an increased risk of osteoporosis [60]. Currently, data on these specific components remain limited and fragmentary, precluding definitive conclusions.

## 6. Bone Quality—Trabecular Bone Score

Several non-invasive techniques have been developed to evaluate bone microarchitecture, an index of bone quality [61]. As a non-invasive, imaging-derived parameter, trabecular bone scores (TBS) offers an indirect assessment of bone microarchitecture, providing valuable insights into fracture risk beyond traditional BMD measurements [61]. TBS evaluates pixel gray-level variations in lumbar spine DXA images, reflecting the texture and quality trabecular bone [62]. Lower TBS values (≤1.350) correspond to deteriorated bone microarchitecture, making individuals more susceptible to fractures [62]. Despite a weak correlation between TBS and direct bone microarchitecture in vivo, TBS has been validated as a reliable predictor of major osteoporotic fractures, especially in postmenopausal women and men over 50 years old [61].

Recent research consistently highlights a significant negative association between MetS and TBS [63]. Although individuals with MetS had a significantly lower prevalence of compromised BMD compared to those without MetS, TBS results showed a different trend. In a cohort of 2380 individuals aged ≥ 60 years, degraded bone microarchitecture (TBS ≤ 1.2) was significantly more prevalent among men and women with MetS after an age-adjusted analysis [57]. Similarly, postmenopausal women with BMI ≥ 35 kg/m^2^ have shown significantly lower TBS values, indicating poor bone architecture despite increased BMD [64].

Crucially, obesity-related metabolic alterations, especially those linked to visceral obesity, exacerbate bone fragility despite seemingly normal or even increased BMD [65]. A compelling study by Totaro et al. investigated the relationship between MetS and TBS, emphasizing WC as a key predictor of bone quality degradation [14]. Analyzing data from 3962 women in the 2005–2008 NHANES cohorts, they found a significant inverse correlation between TBS and various MetS components, with WC showing the strongest association [14]. Even after adjusting for confounding factors such as age, vitamin D levels, smoking, and insulin resistance, WC emerged as the best predictor of degraded TBS. Moreover, increased WC was significantly associated with a higher risk of bone fractures, particularly in women under 50 years old [14]. In addition, other studies corroborate these findings, indicating that WC, rather than BMI, is the strongest predictor of degraded TBS in overweight/obese with MetS, independent of BMD [13,57]. This strongly suggests that visceral obesity negatively impacts bone microarchitecture rather than bone quantity, independent of other metabolic factors.

As mentioned above, our current understanding of the precise impact of hypertension and dyslipidemia, both common components of MetS, on osteoporosis remains limited. To our knowledge, no studies have specifically examined the influence of these MetS components on TBS. Therefore, further research is needed to establish a clear correlation between dyslipidemia and hypertension and their effect on bone quality.

In conclusion, these findings suggest that MetS exerts a detrimental effect on bone quality, predominantly through the influence of visceral obesity. While BMD remains a critical parameter for assessing skeletal health, TBS offers valuable complementary information, particularly in individuals with MetS, who may have normal or elevated BMD but compromised bone microarchitecture.

## 7. Fragility Fractures

Fragility fractures, resulting from low-energy trauma, are a critical indicator of osteoporosis, often being the first sign of compromised bone health [66]. The impact of MetS on fracture risk has been extensively studied [57]. However, drawing definitive conclusions remains challenging due to the heterogeneity across research, including differences in population groups (ethnicity, gender, and age), fracture sites examined, follow-up durations, and MetS definitions. Some studies have reported a reduced fracture risk among individuals with MetS compared to those without it [67,68]. For instance, the Tromsø Study found that individuals with MetS had a decreased risk of non-vertebral fractures, particularly in men with hypertension and in women with increased BMI [67]. Conversely, other studies have indicated an increased fracture risk associated with MetS, especially in women [69,70].

Each component of MetS appears to exert a distinct influence on fracture risk. For example, while IR and hyperglycemia are associated with increased BMD, they paradoxically appear to be associated with an higher fracture risk [70]. Variations in lipid profiles are also associated with fracture risk [71].

Among all MetS components, obesity appears to have the most significant impact on fracture risk. Recent epidemiologic studies challenge the longstanding belief that obesity protects against fractures, showing instead that individuals with obesity are at an increased risk of fractures, despite a normal or above normal BMD [72,73]. The heightened fracture risk of obesity is secondary to mechanical loads, increased risk of fall, and, critically, excess of visceral adiposity. The fracture occurrence in patients with obesity has a specific site distribution in both sex [74]. Compared to women of typical weight or underweight, obese women generally experience fewer hip and wrist fractures but more ankle and lower leg fractures; some studies also report an increased risk of humerus fractures [70,75]. In a large population-based cohort study of older men, obesity was associated with an increased frequency of multiple rib fractures but a reduced risk of hip, clinical spine, wrist, and pelvic fractures [76]. However, when body composition is analyzed rather than just BMI, the osteoprotective effect regarding hip fractures seems to disappear. Indeed, a meta-analysis of seven studies involving 551,224 individuals found that waist circumference and waist-to-hip ratio positively correlate with an increased risk of hip fracture [77]. For vertebral fractures, the data are conflicting and differ according to sex [78,79]. However, recent studies suggest a positive association between BMI and prevalent morphometric vertebral fracture assessed on conventional radiographs [78] and an increased risk of vertebral deformities (not clinically diagnosed) determined by vertebral fracture assessment or morphometric DXA [79].

These findings reinforce the notion that obesity cannot be considered a protective factor against fragility fractures [72]. The site-specificity fractures observed in obese individuals (ankle and proximal humerus) indicate that they are primarily traumatic fractures resulting from increased mechanical stress due to excess body weight, rather than typical fragility fractures [72,75]. This idea is further supported by the distinct presentation of hip fractures in obese individuals with central fat distribution, suggesting that different metabolic obesity phenotypes are associated with different bone health characteristics.

In summary, the relationship between MetS and bone fracture risk is not yet fully elucidated. However, most available studies point towards a negative association between visceral adipose tissue or hyperglycemia and fracture risk, suggesting that patients with these characteristics may face a higher risk of fractures.

In Table 3, we have summarized the evidence regarding the evaluation of bone health in patients with MetS.

## 8. Conclusions

Understanding the specific effects of each component of MetS on bone health is crucial, albeit remains challenging. Our knowledge of the pathophysiology of MetS has been evolving over the years, with new evidence appearing to involve previously little-explored areas, such as the role of the intestinal microbiota and microRNAs [80,81]. Generally, MetS is associated with higher BMD, but this quantitative benefit hides a critical paradox: MetS appears to exert a detrimental or negligible impact on bone quality, as assessed by TBS, largely driven by BMI, WC, and dysglicemia.

Further research is necessary to clarify these paradoxical effects and to assess the overall impact of MetS on bone health and fracture risk. Given the compelling evidence, a proactive approach to assessing fracture risk is crucial for patients with MetS with impaired glucose metabolism and visceral obesity, who face a significantly higher risk of fractures.

## Figures and Tables

**Table 1:** Metabolic Syndrome diagnosis according to the revised National Cholesterol Education Program Adult Treatment Panel III (NCEP-ATP III) criteria.

Diagnosis of MetS if ≥3 of the Following Criteria:	
Waist Circumference	≥102 cm in men ≥88 cm in women
Triglycerides	≥150 mg/dL
HDL Cholesterol	<40 mg/dL in men <50 mg/dL in women
Blood Pressure	≥130/85 mmHg or on antihypertensive medications
Fasting Glucose	≥100 mg/dL or on antidiabetic treatment

MetS = Metabolic Syndrome.

**Table 2:** Evidence from meta-analysis on fracture risk associated with current pharmacological interventions for metabolic syndrome.

Drugs	Fracture Risk
**Antidiabetic Drugs**	
Metformin	Reduction [26]
Thiazolidinediones (PPAR-γ agonists)	Increase [26]
Sulfonylures	Neutral [26]
GLP-1 receptor agonists	Reduction [27]
GLP-1/GIP receptor agonists	No data available
DPP-4 i	Neutral [27]
SGLT2 i	Neutral [31]
Insulin	Neutral? [26]
**Antihypertensive drugs**	
Loop diuretics	Increase [32]
Thiazide diuretics	Reduction [32]
Beta-blockers	Reduction [30]
**Hypolipidemic drugs**	
Statins	Reduction [29]
Ezetimibe	No data available
Bempedoic acid	No data available
PCSK9 i	Neutral [28]

Legend: PPAR-γ, peroxisome proliferator-activated receptor gamma; GLP-1, glucagon-like peptide-1; GIP, glucose-dependent insulinotropic polypeptide; DPP-4, dipeptidyl peptidase 4 inhibitors; SGLT2 i, sodium-glucose cotransporter-2 inhibitors; PCSK9 i, proprotein convertase subtilisin/kexin type 9 inhibitors.

**Table 3:** Bone health assessment in patients with Metabolic Syndrome. Legenda: MetS, metabolic syndrome; Ca, calcium; P, phosphate; hypo vit D, Hypovitaminosis D; U-Ca, urinary calcium; BMD, bone mineral density; TBS, trabecular bone score; WC, waist circumference. The ↑ symbol indicates an increase, and ↓ indicates a decrease.

Parameter	Role in Bone Health Evaluation of MetS Patients
**Bone turnover**	Potentially useful but with uncertain predictive value in assessing fracture risk; Especially hyperglycemia and obesity, often leads to suppression of both bone formation and resorption.
**Ca-P homeostasis**	The common disruption of mineral homeostasis is linked to impaired bone remodeling and increased skeletal fragility; Typical Ca-P dysregulation in MetS: ↓ Ca; ↓ P; ↓ hypo vitD; ↑ U-Ca.
**Bone mass (BMD)**	A relative BMD deficiency and abnormal bone microarchitecture is frequent, similar to other secondary osteoporosis; Especially hyperglycemia and abdominal obesity may have a preserved BMD, with higher fracture risk.
**Bone quality (TBS)**	Bone quality is typically impaired; TBS offers complementary information, especially for MetS individuals with normal BMD; WC is a key predictor of bone quality degradation, showing the strongest association among MetS components.
**Fragility fractures**	The relationship between MetS and fragility fractures is complex and inconsistent across studies; Obesity increases fracture risk—especially at ankles and lower legs—despite potentially normal or high BMD.
